# Organic geochemistry of the middle Paleozoic Ora Formation in Iraq: Implications for source rock assessment and hydrocarbon potentiality

**DOI:** 10.1016/j.heliyon.2024.e29782

**Published:** 2024-04-18

**Authors:** Al-Auqadi Rahma S, Mohamed W. Alkhafaji, Ali I. Al-Juboury, Alex Zumberge, Nasir Alarifi, Dan Jarvie, Giovanni Zanoni, Harry Rowe

**Affiliations:** a College of Petroleum and Mining Engineering, University of Mosul, Iraq; b Department of Applied Geology, College of Science, University of Tikrit, Iraq; c Al-Kitab University, Kirkuk, 36015, Iraq; d GeoMark Research, Houston, TX, USA; e Geology and Geophysics Department, College of Science, King Saud University, Riyadh, Saudi Arabia; f Worldwide Geochemistry, LLC, Houston, TX, USA; g RohmTek, Houston, TX, 77024, USA; h Premier Corex Laboratories, Houston, TX, 77041, USA

**Keywords:** Ora formation, Middle Paleozoic, Source rock potentiality, Light hydrocarbon, Molecular geochemistry, Mineralogy, Iraq

## Abstract

The Ora Formation (late Devonian–early Carboniferous) is thought to be a potential source rocks for the Paleozoic petroleum system of Iraq. The source potential from the Ora Formation is evaluated for the first time ever in this study from western and northern Iraq which integrates data from organic geochemistry including Total Organic Carbon (TOC) analysis, HAWK pyrolysis, gas chromatography (GC), and gas chromatography mass spectrometry (GC-MS) and mineralogical X-ray diffraction and scanning electron microscopy. The shale and muddy carbonate succession within the Ora Formation from surface section in northernmost Iraq and subsurface section from two wells (Akkas-1 and Akkas −3) from western Iraq have been employed to assess the source rock potentiality, thermal maturity, kerogen type, organic content, and depositional environment. In addition to organic geochemical analyses, mineralogical XRD and SEM-EDS were used to support the paleoenvironmental interpretation of the Ora Formation. The results from TOC and HAWK analyses reveal that the Ora Formation ranges from poor to good as a source rock. However, the HAWK data suggests that the surface samples from northernmost Iraq are highly mature, highly weathered, or both. Kerogen analysis revealed that the Ora Formation contains immature type III and mixed II-III kerogens. Low TOC values were attributed to factors such as significant clastic input, weathering effects, and the prevailing oxic environment during deposition. The presence of detrital influx of quartz and feldspars, along with the occurrence of illite and kaolinite clay minerals, suggest a detrital input with weathering influence under hot arid and warm humid conditions. Biomarker analysis of the light hydrocarbons using GC and GC-MS revealed that these light hydrocarbons were generated from marine planktonic algae sources, possibly with some contributions from terrestrial and/or microbially reworked organic matter. These high mature light hydrocarbons in subsurface section were originated from anoxic marine shale source rocks. They were most likely from the Cambro-Ordovician Khabour Formation and were contaminated from another source.

## Highlights

• First Assessement of Source Rock Potentiality.
• Light Hydrocarbons.
• Middle Paleozoic, Iraq.
• Biomarker, Pyrolysis, SEM-EDS-SEM.

## 1 Introduction

Global Paleozoic basins have produced about 25 % of the world's oil reserves, making them one of the most important Phanerozoic petroleum source rocks. As a result, these basins are extremely important for improving our comprehension of and evaluation of the global petroleum province in addition to future hydrocarbon reserves [[1], [2], [3]].

Due to the limited extent of pre-Mesozoic exploration in Iraq, there is a scarcity of knowledge regarding the Paleozoic petroleum system [4,5]. The middle Paleozoic Ora Formation is a shallow water tidal, siliciclastic-shale, sandstones and siltstones, and carbonate (dolostones) succession that symbolizes the change from the marine carbonates of the Harur Formation to the clastic-dominated Kaista Formation [6]. The formation is also recognized as one of the organic-rich formations in Iraq in addition to its characteristic sealing capacity [4]. Based on brachiopods *Avonia praelongus, Spirifer julii and Spirifer verneuili*, the late Devonian-early Carboniferous age is determined for the formation [7].

The majority of previous researches on the Ora Formation were concentrated on the entire middle Paleozoic sequence including the Ora Formation in terms of sequence stratigraphy [8], facies and depositional environments [6,9], palynostratigraphy [[10], [11], [12]], and oil and gas generation based on palynology [13]. However, the literature is very restricted regarding information about the petroleum potential and source rocks characterization. The Paleozoic potential source rocks in Iraq include the shales of the Ora Formation as well as the shales of the Khabour and Akkas formations [[14], [15], [16]]. Previous studies have mentioned that the Ora formation's average total organic carbon (TOC) values in the Akkas-1 well and Khleisia-1 well of western Iraq are 1.5 % and 3.5 %, respectively [17]. The high S3/TOC ratios found in the Ora Formation point to more oxic deposition conditions [16].

The organic geochemistry data, Total Organic Carbon (TOC) and HAWK Pyrolysis, were conducted on 52 samples extracted from the middle Paleozoic Ora Formation. These samples were obtained from both Akkas-1 and Akkas-3 wells in western Iraq, as well as the Ora type section in the northernmost part of Iraq. Additionally, selected samples underwent analysis using Gas Chromatography (GC) and gas chromatography mass spectrometry (GCMS). Mineralogical X-ray diffraction (XRD) analysis and scanning electron microscopy (SEM with EDS) also are conducted. This set of analyses provides crucial insights into the nature of organic matter, petroleum generation potential, thermal maturity, and redox conditions within the Paleozoic (late Devonian-early Carboniferous) rocks in Iraq. The study aims to explore the petroleum source rocks and assess the source rock potential of the Ora Formation, particularly in one of the economically significant regions of the Middle East. Additionally, the research aims to identify the possible source of light hydrocarbons in the Akkas field of western Iraq.

## 2 Geological setting

**Fig. 1:**
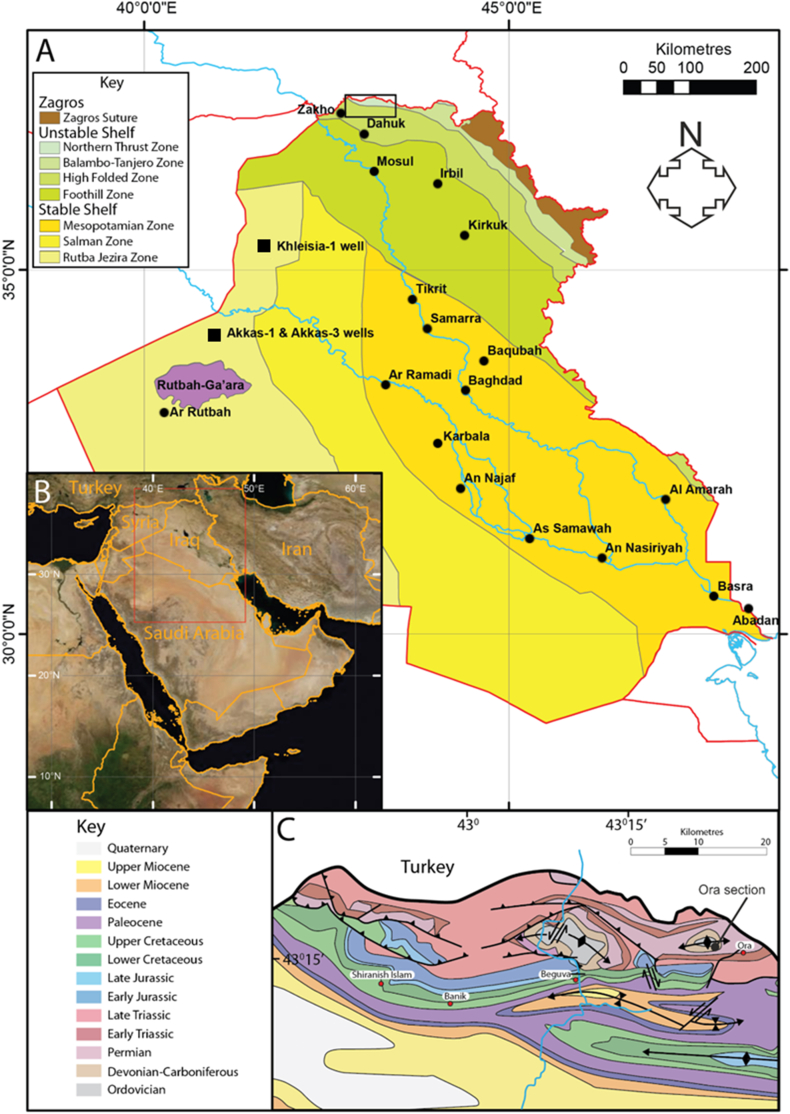
Structural provinces of Iraq modified after [19] showing the location of some wells penetrating Ora Formation. The location of map C is indicated by the black box. B: Inset map shows countries neighboring Iraq; the location of map A is indicated by the red box. C: Geological map of northern Iraq modified after [20] showing the location of the Ora type section. Black rectangular on the top of the map in A, represents the location of the studied surface section. (For interpretation of the references to colour in this figure legend, the reader is referred to the Web version of this article.)

The siliciclastic-carbonate facies of the middle Paleozoic (Devonian–early Carboniferous) succession are exemplified by the Kaista, Ora, and Harur formations in Iraq. This sequence, which represents epicontinental or epeiric seas within a homoclinic carbonate ramp, is believed to have been formed in a subsiding basin [6,9,14,18]. The Ora Formation is found in various wells in western Iraq, including Akkas-1, Akkas-3, and Kheisia-1 (Fig. 1), and it is exposed in the northernmost part of the country.

Intracratonic basin development during the Devonian–Early Carboniferous period is thought to have resulted from compression and extension due to the Hercynian orogeny. (Tectonic Megasequence AP4; [21]). Widespread deposition of marine shale and limestone was recorded in Iraq, Syria and southern parts of Turkey [19,22].

**Fig. 2:**
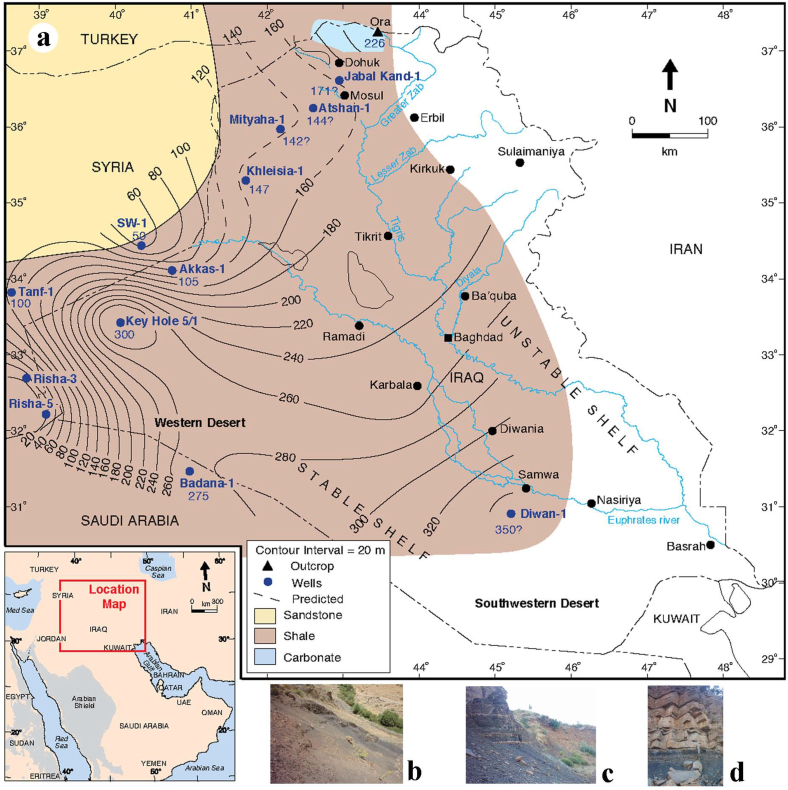
a- Isopach map showing the facies distribution and thicknesses of the Ora Formation in northwestern Iraq [14]. The formation consists predominantly of marine shales with minor carbonates, and towards Syria it becomes dominated by sandstones. b-c, Succession of black shale and thin limestone beds in Ora section. d- Contact between Ora Formation (black shale) and the overlying Harur Formation (thick bedded limestone).

Various thicknesses were recorded for the Ora Formation in subsurface sections at Akkas-1, Akkas-3, (KH) 5/1 and Khleisia-1 wells ranging 105–300 m [17,23]. Ora Formation crops out at several localities with various thicknesses in extreme northern Iraq. The thickness of the formation in the type-section at Ora fold is 226 m [7], (Fig. 2).

The Ora Formation exhibits a distinctive lithology consisting of interbedded shale with subordinate sandstones, siltstones, and dolomitic units. This lithological composition is observed in both surface and subsurface sections (Fig. 2). Throughout most of Iraq, the dominant lithologies within the formation are marine shales with minor occurrences of carbonates. However, in the vicinity of the Ora type area, the carbonates are more extensive. Towards Syria, the formation undergoes a lithological transition, with sandstone becoming the predominant lithology (Fig. 2). The Ora Formation is composed of common black micaceous and calcareous shales [21]. The formation represents the progradation of a siliciclastic ramp onto a shallow to open-marine platform during a regional transgression [14].

Hydrothermal activity most likely had an impact on the Ora Formation's depositional setting, and it may have been connected to the subduction of Paleo-Tethys' southern margin as noted by Ref. [24]. Paleoredox proxies indicate that the deposition occurred under anoxic conditions, with reduced primary productivity playing a role in the preservation of organic matter within the Ora Formation [24].

**Fig. 3:**
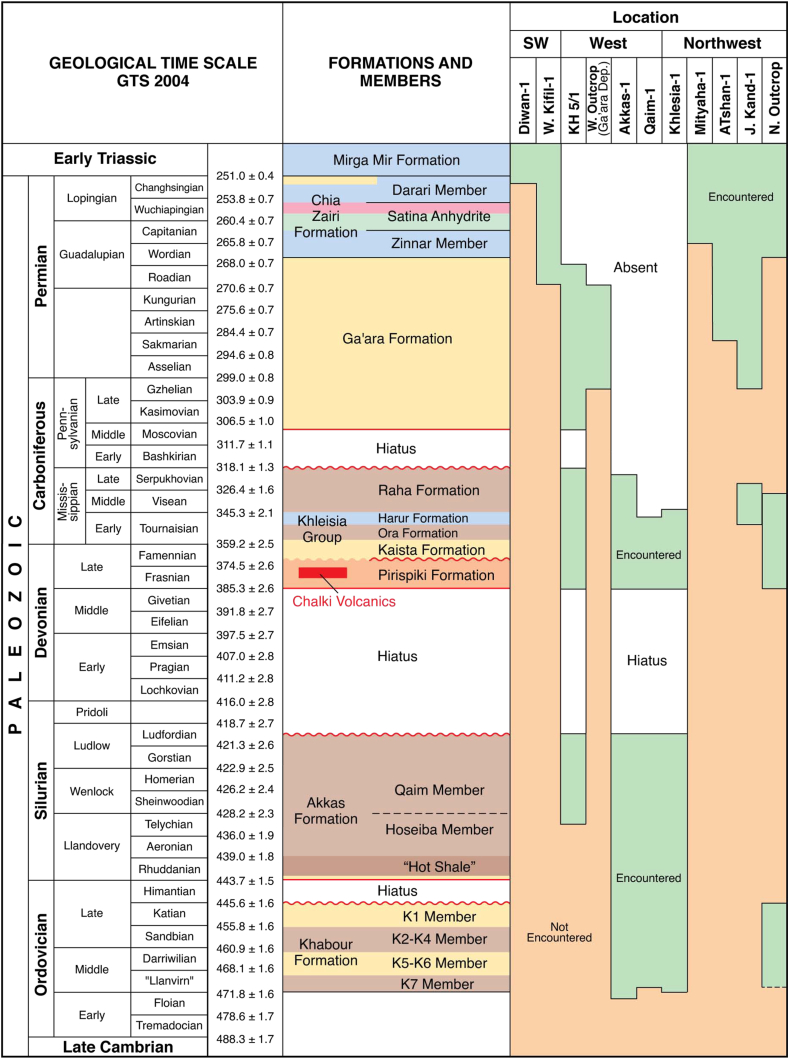
The encountered Paleozoic formations and members of Iraq are shown for the northern and southern outcrops and in wells (green). The intervals shown in orange are where the total depth of the well did not penetrate the formation or the formation does not crop out [14]. (For interpretation of the references to colour in this figure legend, the reader is referred to the Web version of this article.)

The Ora Formation is encountered in outcrops of extreme northern Iraq, whereas it is encountered mainly in subsurface sections from western Iraq, and sometimes encountered in other areas from southwestern and northwestern parts of Iraq (Fig. 3).

## 3 Methodology

**Fig. 4:**
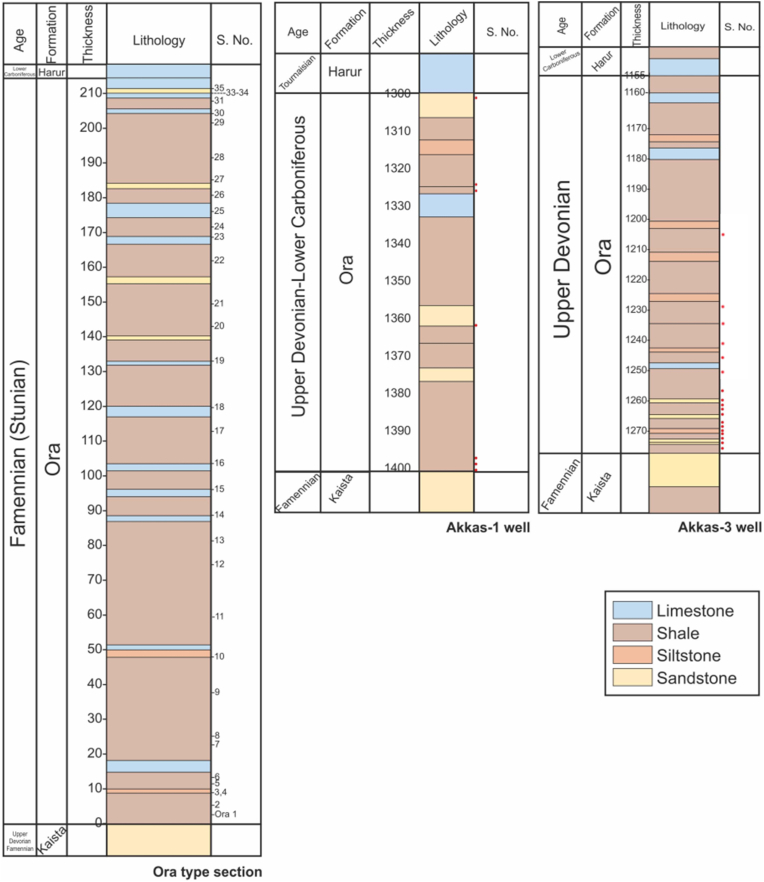
Lithology of the Ora Formation at the studied surface and subsurface sections from northern and western Iraq (see Fig. 1, Fig. 2) with sample locations.

A total of fifty-two samples from both surface section (34 samples) at the Ora locality from extreme northern Iraq and core samples from two subsurface sections in two wells, the Akkas-1 (3 core samples) and Akkas-3 (15 core samples) in western Iraq (Fig. 4) were subjected to organic geochemical analysis (TOC and HAWK pyrolysis). Samples mostly taken from shale units that dominate the lithology of the Ora Formation in addition to sporadic muddy limestone, siltstone and rare sandstones (see Fig. 4).

### 3.1 Total organic carbon

A LECO C230 equipment is used to determine the TOC content which involves the decarbonation of the rock specimen through treatment with hydrochloric acid. This procedure consists of subjecting the weighed samples to concentrated HCl for a duration of 2 h. After that, the filter is put into an LECO crucible and dried for 4 h at 110 °C in a low-temperature oven. Following this, the samples are reweighed to calculate the percentage of carbonate based on the weight loss. The LECO instrument is standardized using a reference material. Every ten measurements, this reference material is examined as an unknown sample to evaluate the analysis's accuracy and consistency. Retests that are both random and specific are carried out to verify the data. A difference of 3 % from the specified reference value is the allowable range for the TOC standard deviation.

#### 3.1.1 HAWK pyrolysis

Samples underwent analysis using the Wildcat Technologies HAWK pyrolysis instrument to determine yields of oil, kerogen, and organic carbon oxides. An approximate 70 mg of whole rock sample was subjected to analysis using standard analytical procedures. The results obtained were then combined with TOC to compute the hydrogen index (HI) and oxygen index (OI). HAWK pyrolysis is a swift analytical technique that offers crucial insights into the oil and kerogen content, thermal maturity, and kerogen type. It estimates the quantity of petroleum produced during the thermal decomposition of kerogen by the HAWK S2 parameter, which is expressed in mg Hc/g. The parameters derived from HAWK pyrolysis encompass S1 (volatile oil pre-pyrolysis), S3, or organic carbon dioxide released when kerogen is thermally cracked, and Tmax, or the highest temperature at which S2 generation peaks. Table (1) provides specifics on these basic parameters that were acquired by HAWK pyrolysis.

### 3.2 Gas chromatography

Bitumen extraction from six subsurface samples was carried out using Dichloromethane (DCM) as a solvent. After 24 h’ magnetic stirrer the solution then was filtered and concentrated. The concentrated solution was separated into saturated hydrocarbons, aromatic hydrocarbons, and resin (NSO compounds) through column chromatography. A Fisons Instruments GC 8000 series ECD 850 with hydrogen as the carrier gas is used for GC analysis. The oven temperature program ranged from 80 °C for 5 min to 300 °C for 20 min at a rate of 5 °C per minute. While Agilent mass spectrometer is used to analyze the saturated fraction from three representative subsurface samples through GC-Mass Spectroscopy. Geomark Research Ltd (Houston, Texas), USA, conducted all the aforementioned analyses.

### 3.3 X-ray diffraction (XRD)

X-ray diffraction mineralogy analysis was conducted at the Premier Corex Laboratory in Houston, Texas, U.S.A. on bulk rock samples (16 surface and 13 subsurface) utilizing a Bruker D8 Advance XRD device with a CuKα radiation. The TOPAS software package is used for quantitative determination of the mineralogical phases in the studied samples.

### 3.4 Scanning electron microscopy (SEM)

The SEM analysis was conducted at the Premier Corex Laboratory in Houston, Texas, U.S.A. using FEI Quanta FEG 650 FE-SEM equipped with Bruker EDS XFlash 5030 and a FEI R580 Everhart-Thornley electron detectors. A total of 6 surface and 4 subsurface samples were used. Samples were mounted on aluminum stubs and coated with 10 nm of Iridium using a Leica EM ACE600 sputter coater. Imaging in secondary and backscattering modes is achieved at 5 μm, 10 μm, 25 μm, 50 μm, 100 μm, 200 μm, and 500 μm field widths.

## 4 Results and discussion

### 4.1 Source rock potential and type of kerogen by TOC and HAWK pyrolysis analysis

Fifty-two shales and muddy limestone samples from surface and subsurface sections were analyzed. Thirty-four samples from the surface section were collected from extreme northern Iraq and eighteen core samples from the studied wells from western Iraq. TOC and HAWK pyrolysis parameters are listed in (Table 1).

**Table 1:** HAWK Pyrolysis from the studied surface and subsurface sections of the Ora Formation (see Fig. 4 for sample locations).

Sample number	Sample type	TOC (wt%)	Percent Carbonate	S1 (mg HC/g)	S2 (mg HC/g)	S3 (mg CO2/g)	Tmax (°C)	HI (S2x100/TOC)	OI (S3x100/TOC)	PI (S1/(S1+S2)
Ora 1	outcrop	5.00	5.80	0.51	0.75	0.98	354	15	20	0.40
Ora 2	outcrop	0.47	16.81	0.09	0.02	0.35	338	4	75	0.82
Ora 3	outcrop	3.86	6.62	0.10	0.04	0.43	352	1	11	0.71
Ora 4	outcrop	0.52	10.28	0.05	0.01	0.28	335	2	54	0.83
Ora 5	outcrop	3.95	11.15	0.08	0.03	0.43	345	1	11	0.73
Ora 6	outcrop	1.00	18.70	0.06	0.02	0.49	349	2	49	0.75
Ora 7	outcrop	1.62	15.50	0.13	0.04	0.50	305	2	31	0.76
Ora 8	outcrop	1.70	11.08	0.16	0.08	0.47	313	5	28	0.67
Ora 9	outcrop	0.88	16.94	0.10	0.02	0.44	312	2	50	0.83
Ora 10	outcrop	0.90	15.23	0.09	0.03	0.44	313	3	49	0.75
Ora 11	outcrop	0.39	10.81	0.06	0.04	0.18	332	10	46	0.60
Ora 12	outcrop	0.43	10.63	0.08	0.06	0.18	350	14	42	0.57
Ora 13	outcrop	0.42	12.00	0.08	0.02	0.26	369	5	61	0.80
Ora 14	outcrop	0.58	15.23	0.05	0.03	0.35	346	5	60	0.63
Ora 15	outcrop	0.15	78.71	0.03	0.02	0.31	435	14	212	0.60
Ora 16	outcrop	0.55	15.30	0.06	0.05	0.54	347	9	98	0.55
Ora 17	outcrop	0.65	14.48	0.08	0.03	0.23	330	5	35	0.73
Ora 18	outcrop	1.28	61.05	0.12	0.15	0.52	324	12	41	0.44
Ora 19	outcrop	1.19	22.92	0.16	0.14	0.32	334	12	27	0.53
Ora 20	outcrop	0.93	22.30	0.12	0.14	0.39	467	15	42	0.46
Ora 21	outcrop	0.88	34.80	0.07	0.04	0.51	312	5	58	0.64
Ora 22	outcrop	0.93	18.83	0.06	0.03	0.29	363	3	31	0.67
Ora 23	outcrop	0.28	70.98	0.12	0.11	0.43	464	39	152	0.52
Ora 24	outcrop	0.71	21.94	0.05	0.13	0.30	419	18	42	0.28
Ora 25	outcrop	0.06	99.44	0.04	0.03	0.31	451	50	512	0.57
Ora 26	outcrop	0.62	15.01	0.05	0.03	0.40	363	5	64	0.63
Ora 27	outcrop	1.16	16.88	0.09	0.04	0.41	352	3	35	0.69
Ora 28	outcrop	1.09	17.29	0.06	0.03	0.39	335	3	36	0.67
Ora 29	outcrop	0.59	18.03	0.05	0.04	0.30	350	7	51	0.56
Ora 30	outcrop	0.34	47.00	0.08	0.14	0.33	464	42	98	0.36
Ora 31	outcrop	0.93	12.17	0.06	0.06	0.39	352	6	42	0.50
Ora 33	outcrop	0.09	85.21	0.05	0.02	0.31	462	23	354	0.71
Ora 34	outcrop	0.63	10.91	0.04	0.02	0.39	343	3	62	0.67
Ora 35	outcrop	0.17	90.29	0.06	0.13	0.19	438	75	110	0.32
Akkas-1	Core 1329 m	1.41	32.75	1.72	1.41	0.52	433	100	37	0.55
Akkas-1	Core 1408 m	1.12	18.30	0.80	1.07	0.56	432	96	50	0.43
Akkas-1	Core 1416 m	0.92	25.07	0.35	0.77	0.61	430	84	66	0.31
Akkas-3	Core 1258 m	3.12	12.35	6.64	5.21	0.48	426	167	15	0.56
Akkas-3	Core 1260 m	0.73	24.43	3.18	2.83	0.17	415	390	23	0.53
Akkas-3	Core 1260.5 m	0.66	7.71	1.76	1.72	0.26	412	259	39	0.51
Akkas-3	Core 1263 m	0.72	11.27	3.03	2.41	0.22	418	336	31	0.56
Akkas-3	Core 1264 m	0.20	11.45	0.35	0.49	0.21	424	249	107	0.42
Akkas-3	Core 1266 m	1.22	16.62	2.49	1.11	0.33	420	91	27	0.69
Akkas-3	Core 1266.5 m	0.62	11.47	2.37	1.63	0.16	415	261	26	0.59
Akkas-3	Core 1269 m	3.15	18.07	7.83	4.70	0.49	431	149	16	0.62
Akkas-3	Core 1270 m	1.13	10.63	13.68	1.53	0.21	305	135	19	0.90
Akkas-3	Core 1270.8 m	0.36	12.30	3.10	0.58	0.22	317	160	61	0.84
Akkas-3	Core 1272 m	0.08	8.28	0.10	0.08	0.27	419	95	321	0.56
Akkas-3	Core 1275 m	1.48	12.34	4.75	2.31	0.27	427	156	18	0.67
Akkas-3	Core 3759 m	3.65	18.98	17.28	5.26	0.46	428	144	13	0.77
Akkas-3	Core 3789 m	3.73	11.40	17.11	6.50	0.34	425	174	9	0.72
Akkas-3	Core 3810 m	3.68	16.48	20.34	5.67	0.43	428	154	12	0.78

TOC values range from (0.06–5.00 wt%) in the surface section and in the range of (0.92–1.41) and (0.08–3.73) in Akkas- 1 and Akkas- 3, respectively (Table, 1). According to Ref. [25], the studied surface and subsurface samples are considered poor to excellent source rocks.

Ora Formation carbonate contents (CC) are highly variable ranging from (5.80 to over 99 wt %), (average, 27.95 wt %) and a standard deviation of 26.54 in the surface section, and ranging from (18.30–32.75 wt %) and (7.71–24.43) in subsurface sections of Akkas- 1 and Akkas- 3, respectively, with an average value of (25.37 and 13.59 wt%) and a standard deviation of (7.23 and 4.47), (Table 1).

The HAWK yields are indicated in Table (1). In general, the S1/TOC x100 or normalized oil contents are often very low in surface and subsurface samples. This may result from evaporation of volatile petroleum constituents, alteration due to weathering (oxidation), or simply an anomaly due to the low values for S1 and TOC. However, samples in the Akkas-3 well show oil crossover indicated by values of over 100 mg oil/g TOC [26]. In addition, the relatively low Tmax values (average 411 °C) and they have a relatively high production index (PI) values (0.61) may indicate their immature state. This clearly indicates contamination by hydrocarbons from oil-based drilling mud similar to those recorded in the study on the Cambro-Ordovician shale in the same region [27] or possibly producible petroleum. The analyzed samples were core samples; therefore, the contamination by oil-based drilling mud is ruled out, but the possibility of contamination by migrated hydrocarbons is most likely. Akkas field contains light hydrocarbons, condensates, and gas [17].

The average S2 (mg HC/g rock) is 0.08 in the surface section as well as 1.08 and 2.80 in the subsurface sections Akkas- 1 and Akkas-3 respectively. Hydrogen Indices (HI,” S2x100/TOC”) range between 1 and 50 mg HC/gTOC in the surface samples; and from 84 to 100 and 91–390 mg/g in the Akkas-1 and Akkas-3, respectively (Table 1). Additionally, the oxygen index (OI,” S3x100/TOC”) ranges from 11 to 512 mgCO_2_/gTOC in the surface samples and from 37 to 66 and 9–321 mgCO_2_/gTOC in Akkas-1 and Akkas-3, respectively (Table 1).

**Fig. 5:**
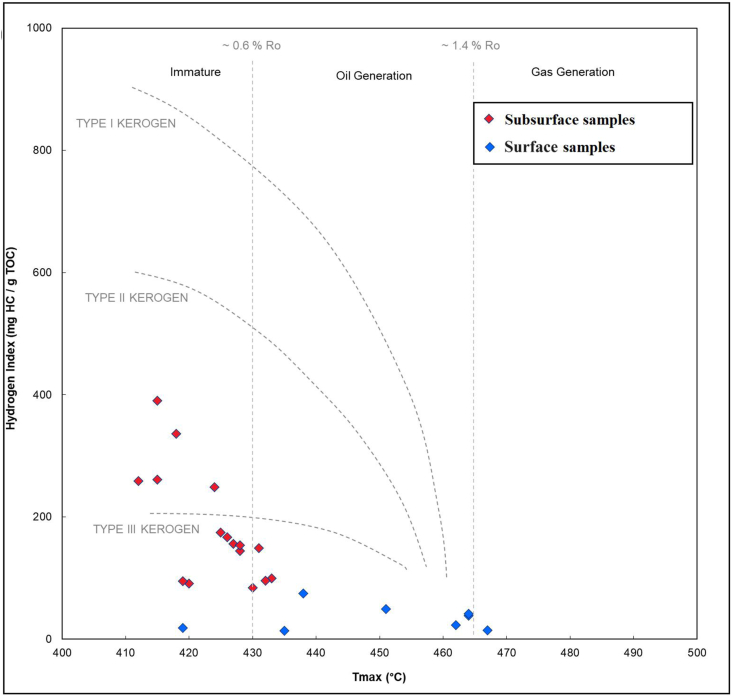
Hydrogen index (HI) versus Tmax diagram for samples from both surface and subsurface sections from the Ora Formation [28]. Number of surface section points is less than in Table (1) due to overlap in the values.

**Fig. 6:**
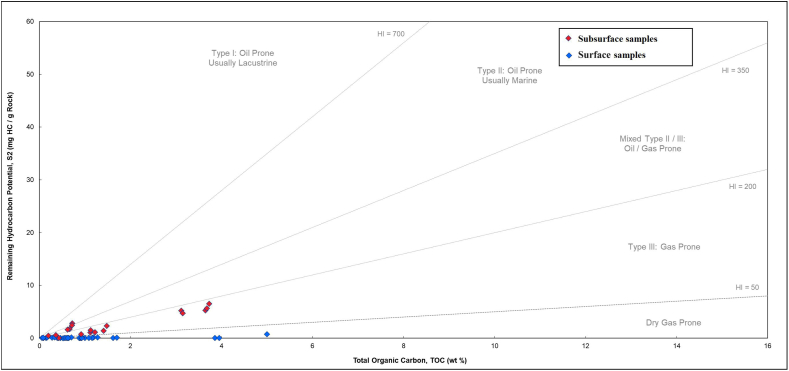
S2 versus TOC diagram for the Ora samples from surface and subsurface sections [29].

The low yields for S2-pyrolysis in the surface section do not yield reliable Tmax values for the kerogen for any of the analyzed samples. Such low yields do not result in a definitive peak from which Tmax is derived. However, based on the low yields, the samples are either highly mature, highly weathered (oxidized), or both. The relatively high oxygen indices suggest that the samples are oxidized. In subsurface sections, the hydrogen index (Fig. 5) and S2 vs TOC (Fig. 6) suggest that the kerogen is predominantly type III, with a few samples displaying a combination of type II/III. The HI versus Tmax cross plot is a standard method for identifying kerogen type in immature organic material [28]. While all surface samples from the Ora Formation belong to type III kerogens, some subsurface samples are categorized as type II-III kerogen (Fig. 5).

Additionally, weathering led to a shift in the kerogen type from type III and mixed II/III in subsurface samples to mainly IV types in surface samples [16] (Fig. 6); as a result of a high degree of thermal maturity or weathering (oxidation), which suggests the existence of inert organic materials. The maturity level is difficult to assess based on Tmax values because most of the studied surface samples have low S2 values (<0.1 mg HC/g rock) which is insufficient for reliable Tmax [25,30]. However, the subsurface samples of the Ora Formation are immature in western Iraq [15,16]. Moreover, no high maturity level is evidenced in the region, therefore, the presence of type IV kerogen is due to oxidation effect [31].

**Fig. 7:**
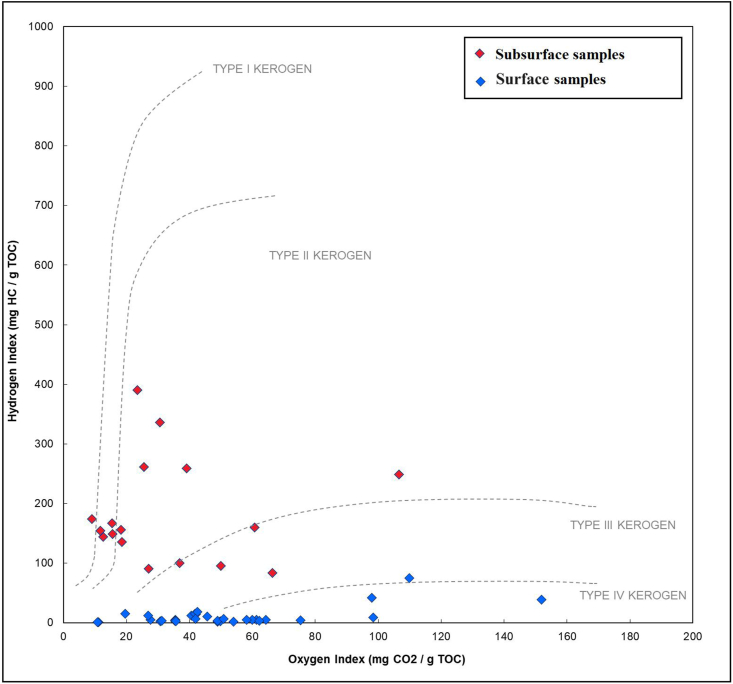
The HI/OI Plot for different kerogen types in the Ora Formation analyzed by rock-pyrolysis [33].

Standard geochemical plots for kerogen type (Fig. 7, HI vs OI) and kerogen type plus thermal maturity (Fig. 5, HI vs Tmax) for surface section are less useful for kerogen typing on these samples. Kerogen-type fields only apply to low-maturity samples [32]. If they are highly mature, the low HI values suggest that the remaining organic matter is strictly gas prone. OI values greater than 200 mg/g indicate either an oxidizing depositional setting or weathering. But in subsurface sections, it is observed that organic matter has high HI (Table 1) (Fig. 7, HI vs OI) and that the organic matter of the Ora Formation in subsurface sections is represented mainly by type III and mixed type ІІ/III (Table 1).

**Fig. 8:**
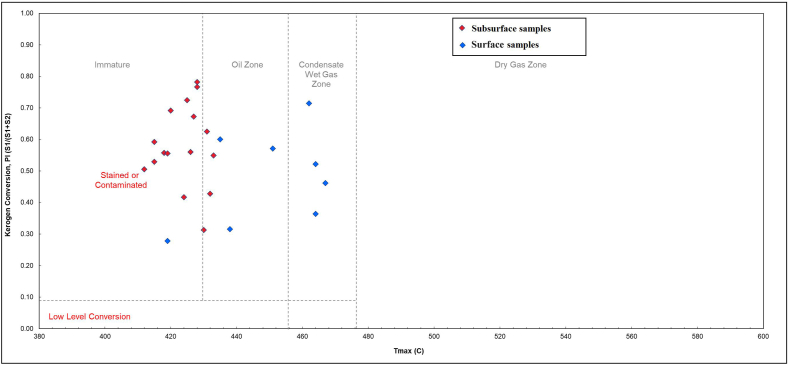
Pyrolysis output plot for source rocks of the Ora Formation based on petroleum potential diagram (PI v Tmax) [34].

Based on petroleum potential diagram (Production Index (PI) versus Tmax) diagram (Fig. 8), the studied surface and subsurface samples vary between immature in the Akkas-3 well to oil zone and condensate dry gas zones in outcrop samples. The high Tmax values of the surface samples are due to oxidation effect which led to reduce the S2 values and give abnormal Tmax values, whereas the high PI values in the subsurface samples are due the contamination effect. In general, pyrolysis data refer to immature or early mature organic matter for the studied samples.

### 4.2 Mineralogical constraints

**Fig. 9:**
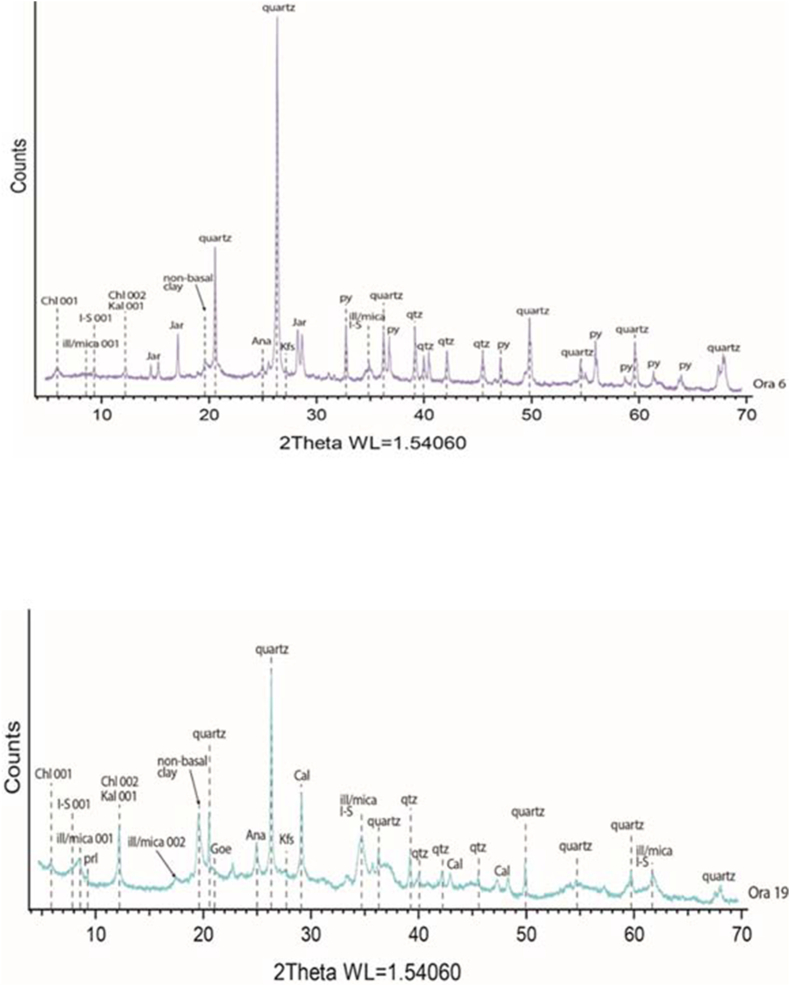
X-ray diffractograms of representative surface samples from the Ora Formation showing various clay and non-clay components. (samples Ora 6 and 19, see Fig. 4 for samples location). Abbreviations; Chl = Chlorite; I–S= Illite-smectite mixed layers; Ill/mica = Illite/mica; Kal = Kaolinite; Jar = Jarosite; Goe = Goethite, Ana = Anatase; Kfs = F-feldspars; qtz = Quartz; Cal = Calcite; Py = Pyrite).

**Fig. 10:**
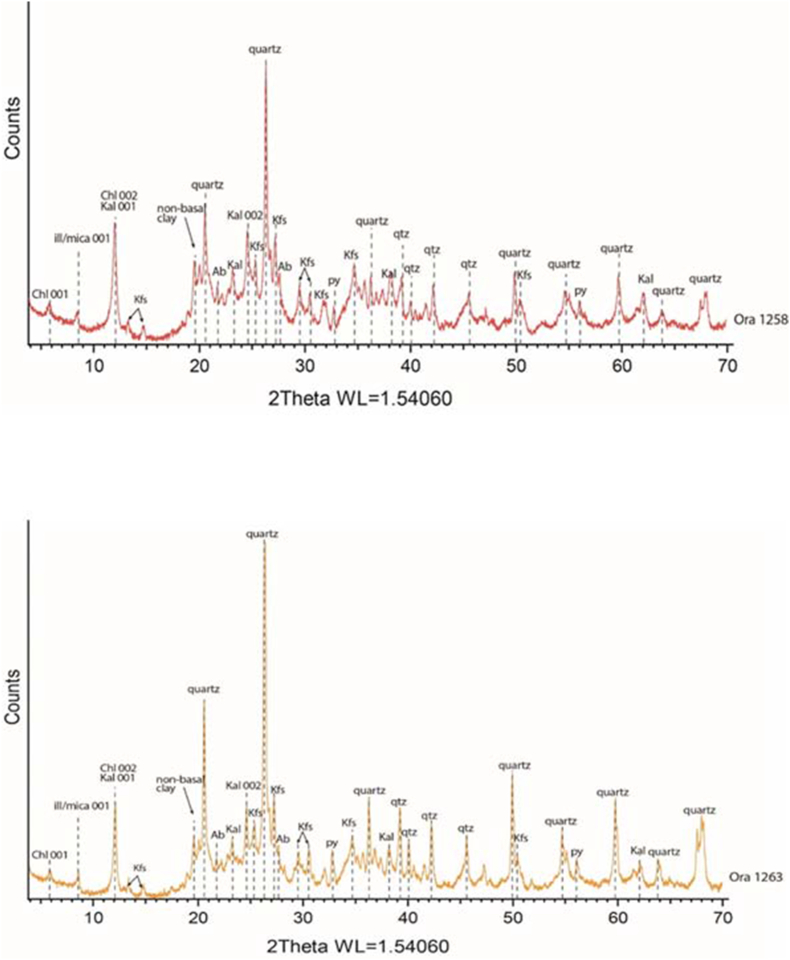
X-ray diffractograms of representative Akkas-3 samples from the Ora Formation showing various clay and non-clay components. (samples at depth 1258, 1263, see Fig. 4 for samples location). Abbreviations; Chl = Chlorite; I–S= Illite-smectite mixed layers; Ill/mica = Illite/mica; Kal = Kaolinite; Ab = Albite; Kfs = F-feldspars; Py = Pyrite; qtz = Quartz.

The Ora Formation exhibits varying TOC values in both surface and subsurface sections, with generally low values observed in the surface sections (Table 1). Several factors contribute to these low TOC values. Initially, the dilution effect occurs when the basin experiences a high influx of clastic input and sedimentation rate, resulting in the formation of organic-poor sediments. The presence of quartz and feldspars as indicated by the mineralogical XRD study conducted in both surface and subsurface sections (Table 2; Fig. 9, Fig. 10), further confirms the detrital influx.

**Table 2:** Mineralogical components of the studied samples from both surface samples (Ora 1-Ora 31) and subsurface samples from Akkas-3 well, Ora 1258 m –Ora 1329 m), (see Fig. 4 for sample locations).Qz = Quartz; Plg = Plagioclase feldspar; K-fel = K-feldspar; Cal = Calcite; Dol = Dolomite; Sid = Siderite; Ana = Anatase; Py = Pyrite; Goe = Goethite; Ml = Mixed layers; I + M=Illite and mica; Ch = Chlorite; Kao = Kaolinite.

Sample number	Framework Silicate			Clay	Carbonate			Others			Total	Clay Minerals				Total
	Qz %	Plg. %	K-fel. %	Clay %	Cal. %	Dol. %	Sid. %	Ana. %	Py. %	Goe./Jar. %		ML I/S %	I + M %	Ch. %	Kao. %	
Ora 31	27.6	1.2	1.4	60.2	2.6	0.9	0.0	1.2	0.0	4.9	100.0	27.8	30.2	1.9	0.3	60.2
Ora 29	18.9	2.9	1.1	58.0	14.4	0.2	0.0	0.7	0.0	3.8	100.0	31.0	21.1	4.8	1.1	58.0
Ora 26	11.8	7.0	3.1	53.7	16.9	0.0	0.0	0.7	0.0	6.8	100.0	34.6	12.6	5.6	0.9	53.7
Ora 24	28.2	6.1	2.3	45.4	13.0	0.8	0.0	0.9	0.0	3.3	100.0	34.3	6.6	3.5	1.0	45.4
Ora 22	22.1	0.4	0.2	57.1	20.0	0.0	0.0	0.0	0.0	0.2	100.0	34.2	17.1	5.7	0.1	57.1
Ora 21	20.2	1.1	1.5	49.8	21.4	0.6	0.0	0.6	0.0	4.8	100.0	33.7	12.3	3.8	0.0	49.8
Ora 20	27.4	2.8	2.0	48.8	10.8	0.0	0.0	1.0	0.0	7.2	100.0	24.0	20.0	2.9	2.9	48.8
Ora 19	26.5	3.5	0.1	58.8	1.2	0.6	0.0	0.8	0.0	8.5	100.0	32.3	24.4	0.0	2.1	58.8
Ora 17	22.9	4.4	2.8	56.1	6.0	0.7	0.0	1.5	0.0	5.6	100.0	22.6	23.3	0.1	10.1	56.1
Ora 13	22.6	6.8	2.0	63.2	0.3	0.0	0.0	1.2	0.0	3.9	100.0	24.7	25.2	4.8	8.5	63.2
Ora 11	36.7	4.2	1.3	47.0	0.5	0.0	0.0	0.6	7.1	2.6	100.0	24.5	18.4	2.4	1.7	47.0
Ora 9	20.9	3.6	0.2	44.5	0.0	0.0	0.0	0.8	0.0	4.5	100.0	17.8	14.6	0.0	12.1	44.5
Ora 7	52.1	2.1	1.2	38.5	4.6	0.0	0.0	0.0	0.0	1.5	100.0	26.7	11.7	0.1	0.0	38.5
Ora 6	38.7	0.8	1.6	47.1	0.8	0.0	0.0	0.9	2.8	7.3	100.0	13.6	18.8	1.4	0.3	34.1
Ora 5	24.6	6.8	0.4	26.5	1.8	0.0	0.0	0.5	4.2	0.8	100.0	4.9	13.7	0.0	7.9	26.5
Ora 1	29.6	5.3	1.7	57.9	4.1	0.0	0.0	0.8	0.0	0.6	100.0	33.0	16.6	0.0	8.3	57.9
Ora 1258 m	25.4	7.2	17.3	46.6	0.0	0.0	1.7	0.9	0.9	0.0	100.0	0.0	15.6	4.6	26.4	46.6
Ora 1260.5 m	78.0	0.8	8.0	5.7	0.1	0.0	2.0	0.4	5.0	0.0	100.0	Trace	2.6	0.8	2.3	5.7
Ora 1260 m	55.0	2.2	10.3	11.8	0.7	19.2	0.2	0.4	0.2	0.0	100.0	Trace	4.4	2.4	5.0	11.8
Ora 1263 m	52.0	4.5	13.3	26.0	0.6	0.0	1.1	1.0	1.5	0.0	100.0	Trace	9.5	2.5	14.0	26.0
Ora 1264 m	90.0	0.8	4.0	2.3	0.5	0.0	1.4	0.0	1.0	0.0	100.0	0.0	0.0	0.0	2.3	2.3
Ora 1266.5 m	61.0	2.6	13.0	14.3	0.2	0.0	4.2	0.4	4.3	0.0	100.0	Trace	3.5	1.2	9.6	14.3
Ora 1266 m	54.3	3.2	14.2	21.9	0.0	0.0	2.7	0.7	3.0	0.0	100.0	0.4	5.0	1.5	15.0	21.9
Ora 1269 m	21.8	5.0	13.5	47.0	0.0	0.0	11.4	1.2	0.1	0.0	100.0	0.0	14.0	6.0	27.0	47.0
Ora 1270.8 m	64.7	0.3	11.5	18.1	0.0	0.0	4.0	0.7	0.7	0.0	100.0	5.0	4.3	Trace	8.8	18.1
Ora 1270 m	49.0	1.9	9.0	37.0	0.0	0.0	1.2	1.7	0.2	0.0	100.0	8.0	16.0	0.0	13.0	37.0
Ora 1272 m	97.5	0.0	0.5	1.9	0.0	Tr	0.0	0.0	0.1	0.0	100.0	0.0	0.0	0.0	1.9	1.9
Ora 1275 m	40.0	9.2	18.5	30.1	0.5	0.0	0.0	1.0	0.7	0.0	100.0	0.0	9.0	3.0	18.1	30.1
Ora 1329 m	11.0	0.9	2.2	12.1	73.4	0.0	0.0	0.2	0.2	0.0	100.0	2.7	4.0	1.4	4.0	12.1

Generally, the low TOC values of Ora Formation indicate deposition under oxic conditions. However, some intervals have moderate TOC values, which may suggest dysoxic-suboxic conditions. This assumption supported by the presence of small pyrite grains in these intervals of the studied subsurface samples (Fig. 10, Fig. 13E) and its alteration products (goethite and jarosite) in the surface samples, which are formed due to weathering of pyrite (Table 2, Fig. 9, Fig. 10). Aqueous oxidation of pyrite by atmospheric O_2_ leads to the creation of acidic environment that permits formation of iron sulfates including jarosite and goethite [35,36]. Therefore, oxidation and hydrolysis of Fe sulfates and Fe sulfides promotes the formation of jarosite and goethite in the studied shales of the surface section, while pyrite is the most stable iron sulfide minerals in anoxic conditions [37,38]. That is formed mainly by reaction of Fe with H_2_S leading to formation of framboidal pyrite due to its extreme insolubility [39]. The results may indicate that the surface samples may have also deposited under anoxic conditions but they were subjected to weathering and oxidation after uplifting.

**Fig. 11:**
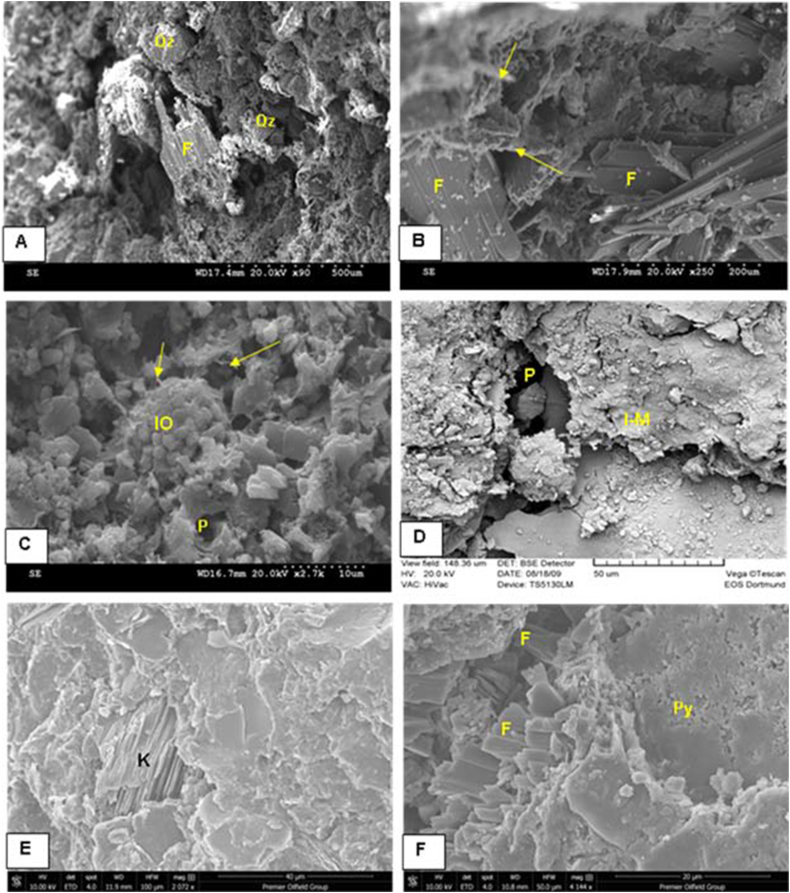
Scanning electron microscopy (SEM) images. A-B, Detrital components of quartz (Qz) and feldspars (F) along with fibers of illite (arrows), sample Ora 4, surface section. C- Cluster of iron oxides (IO), illite fibers (arrows) and pores (P), sample Ora 31, surface section. D- Fractured shale with micro pores (P) and illite-mica (I–M) clay minerals, sample Ora 6, surface section. E-Kaolinite booklets (K), in subsurface shale, sample at depth 1269 mt, Akkas-3 well. F- Feldspar crystals (F) and pyrite grains in subsurface shale, sample at depth 1275 mt, Akkas-3 well. See Fig. 4 for sample locations.

**Fig. 12:**
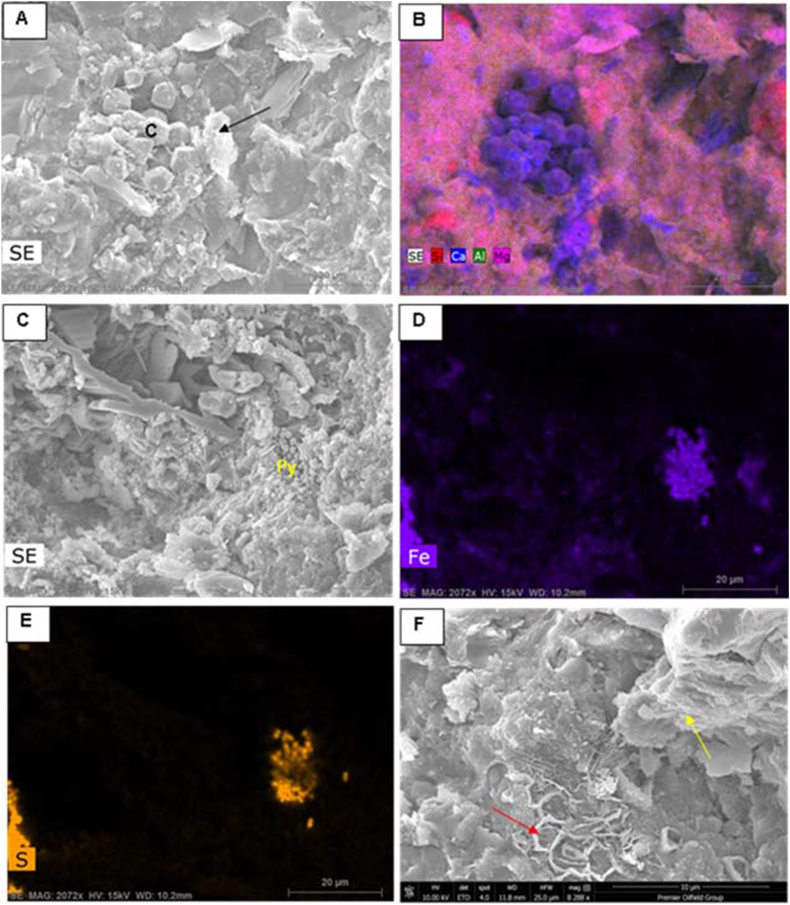
Scanning electron microscopy and energy dispersive spectroscopy (SEM, EDs) images. A- Authigenic pentagonal dodecahedral carbonate (calcite, C) and illite plates (arrow) and their chemical analysis (EDs, B) showing common Ca elemental composition for these carbonates, sample at depth 1269 mt, Akkas-3 well. C-E− Cluster of framboidal pyrite (py) and their elemental composition of Fe and S of pyrite (EDs, D and E), sample at depth 1275 mt, Akkas-3 well. F- network of carbonate composition (red arrow) and flaky illite (yellow arrow) in sample at depth 1269 mt, Akkas-3 well. See Fig. 4 for sample locations. (For interpretation of the references to colour in this figure legend, the reader is referred to the Web version of this article.)

Illite/mica is one of the common clay minerals as indicated from XRD and SEM study (Fig. 9, Fig. 10, Fig. 11, Fig. 12). The existence of illite fibers and illite-mica (Fig. 11 C-D, 12 A, F) alongside kaolinite (Fig. 11 E) suggests a fluctuation in the dry, hot, and humid climatic conditions experienced by the studied Ora samples. This variability could potentially influence their redox conditions [40]. The weathering rate of ancient organic matter is primarily influenced by climate, the source of the organic material, and its maturity [41,42]. Weathering processes, particularly oxidation, can significantly alter the original composition of organic matter at both the bulk and molecular levels. These changes are characterized by reductions in Total Organic Carbon (TOC), extractable organic matter (EOM), and the concentrations of individual compounds [41,43]. In the current investigation, weathering, particularly oxidation, appears to have influenced the examined samples. This could account for the lower TOC values observed in the surface samples compared to the subsurface samples. The average TOC values of the Ora Formation in the surface samples amount to 1.03 %, whereas the subsurface samples exhibit higher average TOC values, measuring 1.5 % in Akkas-1 and 3.5 % in Khlesia-1. It is noteworthy that the organic matter in the subsurface samples has been identified as having a terrigenous origin, as reported by Ref. [17]. Typically, the highest TOC contents in the Ora Formation have low carbonate contents, usually less than 25 wt % (Table 1). This may indicate that terrestrial organic matter is the main source of the kerogen in the Ora Formation.

**Fig. 13:**
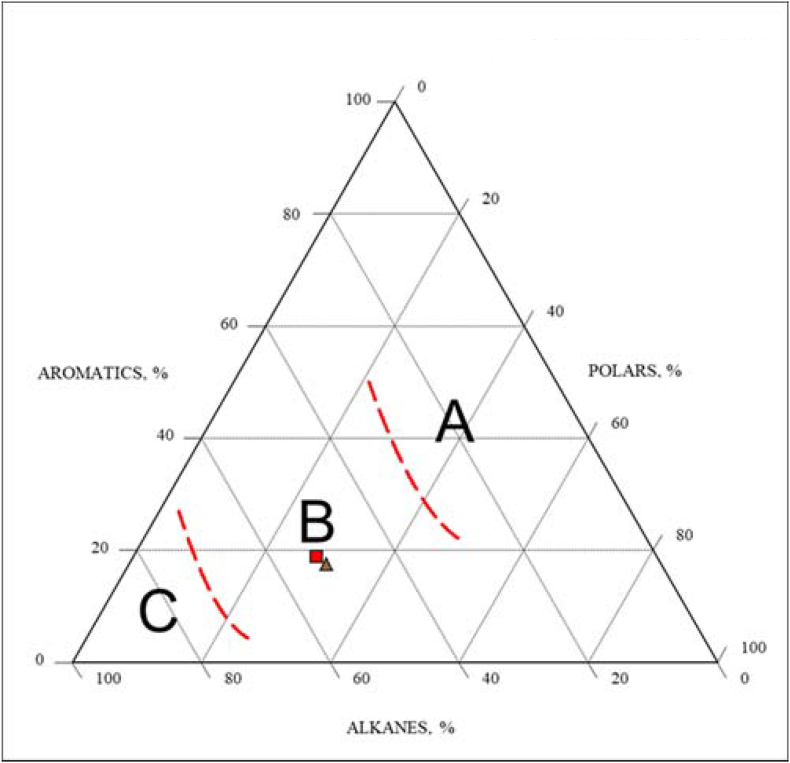
Aromatics-Polars-Alkanes maturity diagram of the studied samples (**A**: Field of immature oils and source rock extracts Ro<0.6 %) (**B:** Field of mature oils and source rock extracts Ro 0.7 %–0.9 %) (**C**: Field of very mature oils Ro>0.9 %) [44].

The Ora Formation samples were analyzed and found to have an average carbonate content (CC) of 28 wt%. Some samples exhibited a high CC, indicating a strong marine or lacustrine influence [45]. On the other hand, other samples showed a low to medium CC, suggesting a marine depositional environment with a significant input of terrestrial material. The low total organic carbon (TOC) and CC values indicate an oxic depositional environment. Additionally, the inverse correlation between TOC and CC (Table 1) suggests that the input of marine organic matter remains consistently low, while terrestrial organic matter is the predominant source. This assumption is supported by high organic index (OI) values and generally low hydrogen index (HI) values (Table 1), indicating a high contribution of terrigenous organic matter. The Ora Formation represents the progradation of a siliciclastic ramp onto a shallow to open-marine carbonate platform during a regional marine regression in the late Devonian, late Famenian period [14,21,46].

### 4.3 Geochemistry of the light hydrocarbon

#### 4.3.1 Bulk composition

**Fig. 14:**
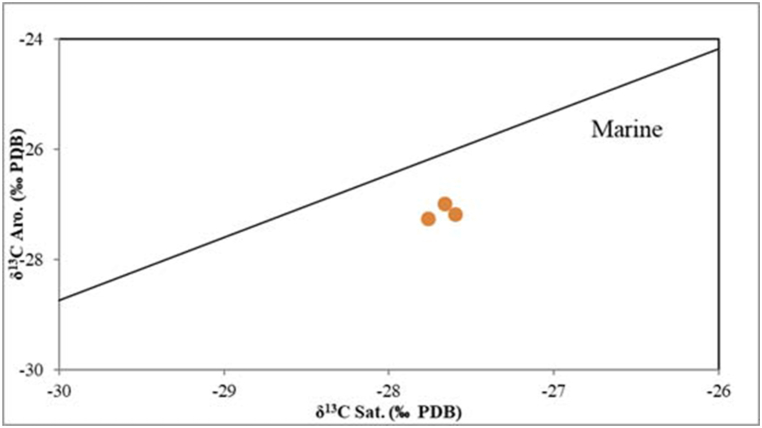
The δ^13^C Saturate versus Aromatic Plot [48].

**Fig. 15:**
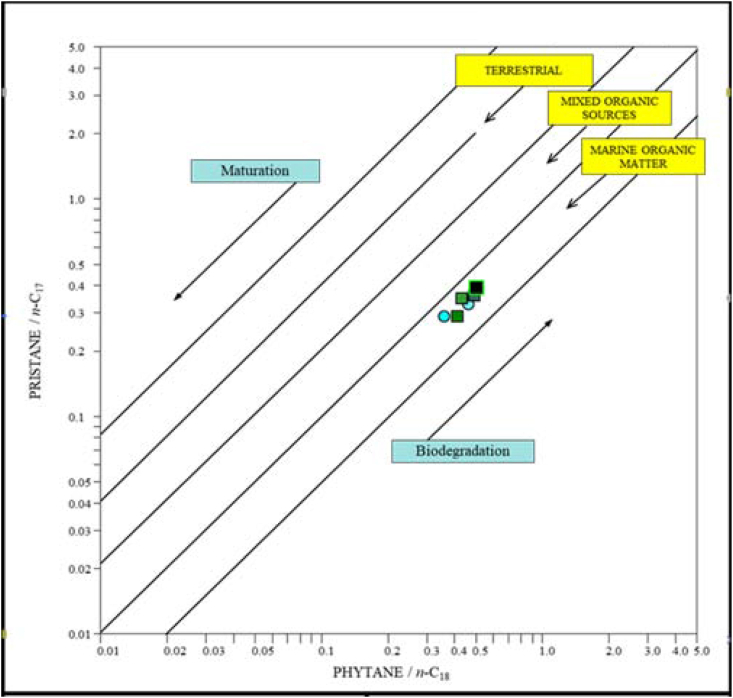
Pr/*n*-C_17_ versus Ph/*n*-C_18_ can be used to infer the type of organic matter in source rocks for the Ora Formation sample [49].

Composition of bulk oil is primarily determined by the type of organic materials, depositional environment, thermal maturity, and alteration processes [34]. As noted above, the rock subsurface samples of Akkas-1 and 3 wells are immature and they are contaminated by hydrocarbons. In all Akkas-3 samples, the asphaltene is very low (0.0–0.6 %) and the saturated and resin components represent the major fractions of the analyzed samples. They range from 50.9 to 52.8 % and 28.2–32.9 % respectively. While the aromatic fractions range from 15.6 to 18.9 % (Table 3, Fig. 13). When the samples are plotted onto a chart [47], all samples plot in the field of mature oils and source rock extracts (Ro 0.7%–0.9 %). The notable prevalence of saturated components over asphaltene in the examined samples strongly supports the notion of maturation. However, this proposition is at odds with the Rock-Eval Tmax values, which indicate a low maturity level for the Ora samples. Consequently, these findings suggest that the extracted bitumen primarily comprises contaminant hydrocarbons, not originating from the Ora Formation. It is essential to highlight that the high silica percentage (97.5) in sample Ora1272 m depth (Table 2) signifies that this sample represents the sandstone unit of the Ora Formation at the Akkas-3 well. Therefore, the hydrocarbons extracted from this sample reflect hydrocarbons that have migrated from a deeper source rock. As a result, the biomarker ratios of this sample distinctly represent migrated hydrocarbons, not originating from the Ora Formation. The δ^13^C isotopes values of the aromatic and saturated hydrocarbons and the canonical variable (CV) values (<−1.5; Table 3) indicate that the analyzed samples are derived from the marine source rock This result contradicts the nature of organic matter in the Ora Formation, which primarily consists of type III kerogen (terrestrial organic matter, Fig. 14). This supports the notion that these isotopic values are indicative of hydrocarbon contamination. The probability of biodegradation is reduced due to the presence of typical alkanes and isoprenoids in the subsurface samples (Akkas-1 and 3 samples). Additionally, the low Pr/*n*-C_17_ and Ph/*n*-C_18_ values (Table 4 and Fig. 15) show that the Akkas-3 samples have not experienced biodegradation.

**Table 3:** Bulk composition and carbon isotope values for the Akkas-3 samples.

Well	Depth (m)	%Sat	%Aro	%NSO	%Asph	%polars	δ 13C saturate	δ 13C aromatic	CV
Akkas-3	3759	51.9	17.5	30.4	0.2	30.6	−27.66	−26.98	−1.57
Akkas-3	3789	50.9	15.6	32.9	0.6	33.5	−27.60	−27.17	−2.14
Akkas-3	3810	52.8	18.9	28.2	0.0	28.2	−27.76	−27.26	−1.93

**Table 4:** Parameter values derived from the distributions and abundances of n-Alkanes and Isoprenoids in the samples of Akkas-1 and Akkas-3.

Well	Depth (m)	Pr/Ph	Pr/*n*-C17	Ph/*n*-C18	CPI	*n*-Alkanes Max.
Akkas-1	1258	0.38	0.39	0.50	1.29	C19
Akkas-1	1269	0.60	0.35	0.43	1.50	C18
Akkas-1	1270	1.06	0.29	0.36	1.64	C17
Akkas-3	3759	0.65	0.33	0.46	1.19	C19
Akkas-3	3789	0.49	0.29	0.41	1.23	C19
Akkas-3	3810	0.65	0.36	0.49	1.17	C19

#### 4.3.2 Source of organic matter and depositional environment of the light hydrocarbons

**Fig. 16:**
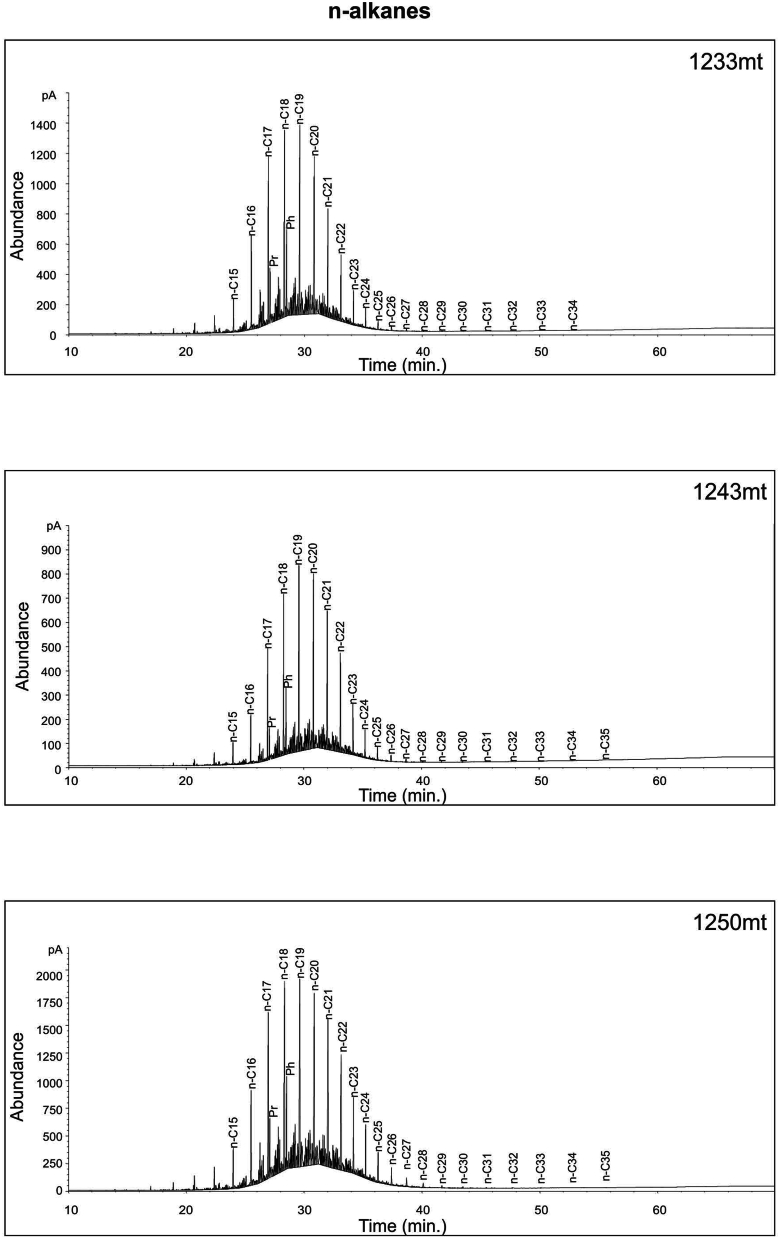
Whole gas chromatograph of selected samples at varies depths from the Ora Formation at Akkas-3 well. See Fig. 4 for sample locations.

**Fig. 17:**
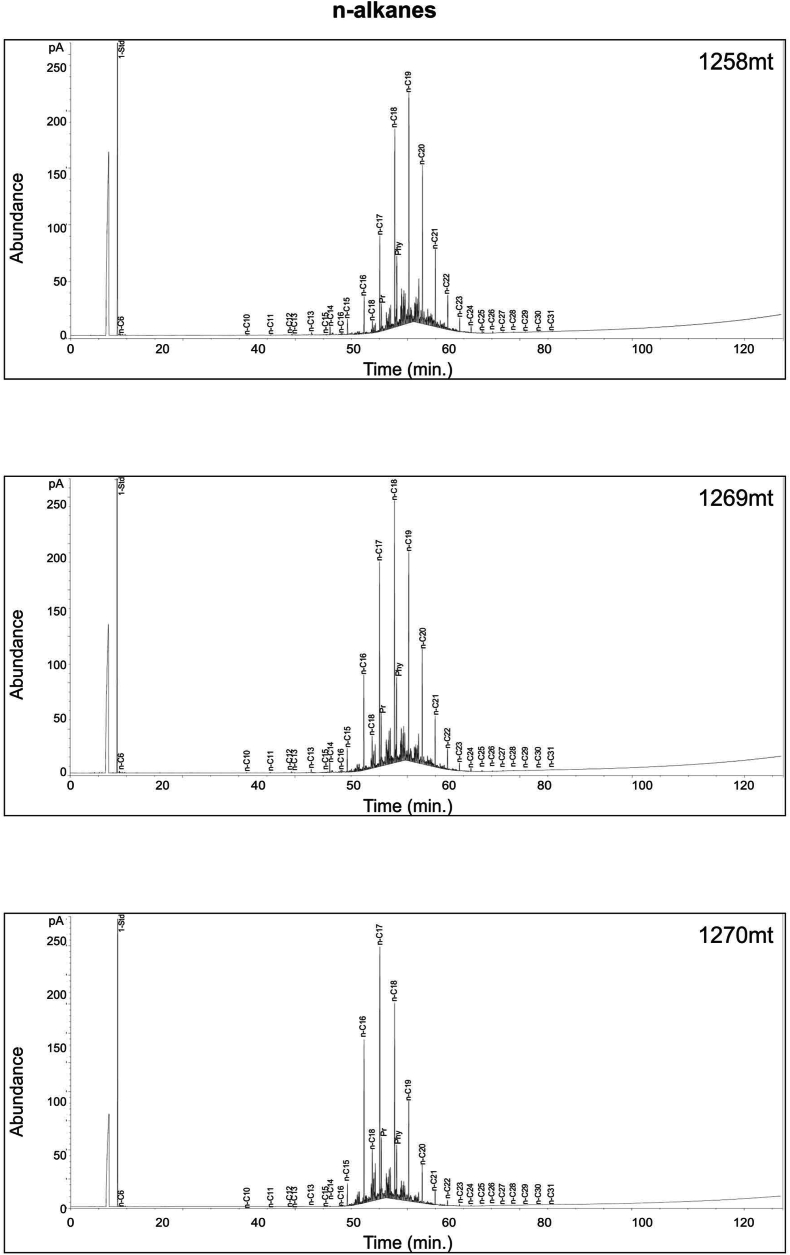
Whole gas chromatographs of selected samples at varies depths from the Ora Formation at Akkas-3 well. See Fig. 4 for sample locations.

Gas chromatograms display a unimodal n-alkane distribution in the samples, with a prevalence of short chains ranging from *n*-C_10_ to *n*-C_35_, maximizing at *n*-C_17_, *n*-C_18_, and n-C_19_ (Fig. 16, Fig. 17). The presence of short-to medium-chain normal alkanes, particularly *n*-C_17_, indicates algal input in source rocks [34,50]. In Paleozoic sediments from upper Ordovician to lower Silurian layers in Iraq [51] and Tunisia [52], a dominance of short-to medium-chain n-alkanes with a peak at *n*-C_16_ or *n*-C_17_a was also observed. The significant concentration of *n*-C_17_, *n*-C_18_, and n-C_19_ alkanes in this study suggests that the organic matter originated from marine planktonic algae. Furthermore, the Pr ∕ *n*-C_17_−Ph∕ *n*-C_18_ ratio in the samples implies that they are marine algal type II kerogens (Fig. 15). The low steranes/17α-hopanes ratio, ranging from 0.59 to 0.71 as indicated in Table 5, suggests the presence of microbially altered marine organic material [53].

**Table 5:** Source –related biomarker ratios for the Akkas-3 samples.

Well	Depth (m)	S/H	C22/C21	C24/C23	C30NH/C30H	Dia/Reg	Ts/Tm	C29/H	C35S/C34S	%C27	%C28	%,C29	DBT/P
Akkas-3	3759	0.59	0.58	0.33	1.19	0.86	0.33	1.19	0.69	45.82	28.10	26.08	1.62
Akkas-3	3789	0.71	0.65	0.42	0.91	0.78	0.39	0.91	0.61	44.84	30.20	24.95	1.47
Akkas-3	3810	0.67	0.63	0.37	1.35	0.75	0.35	1.35	0.59	45.07	28.38	26.55	1.65

**Fig. 18:**
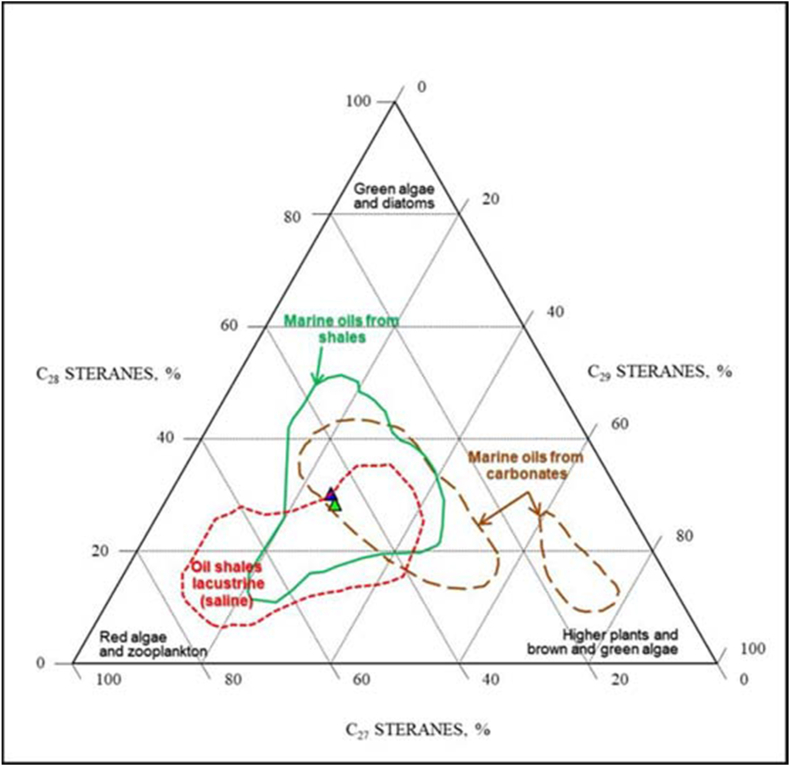
Ternary diagram of relative abundance of C_27_–C_28_–C_29_ regular steranes showing the sources and depositional environment of a sample from the Akkas-3 well, modified from Ref. [60].

The distribution of regular steranes is primarily based on C_29_ steranes, predominantly found in higher plants, brown and green algae, C_28_ steranes found in yeast, fungi, plankton, and algae, and C_27_ steranes found in marine plankton [54,55]. In the current study samples, the elevated C_27_value (Table 5) signifies a substantial contribution of marine algal organic matter (Fig. 18 and Table 5) [[56], [57], [58], [59]].

**Fig. 19:**
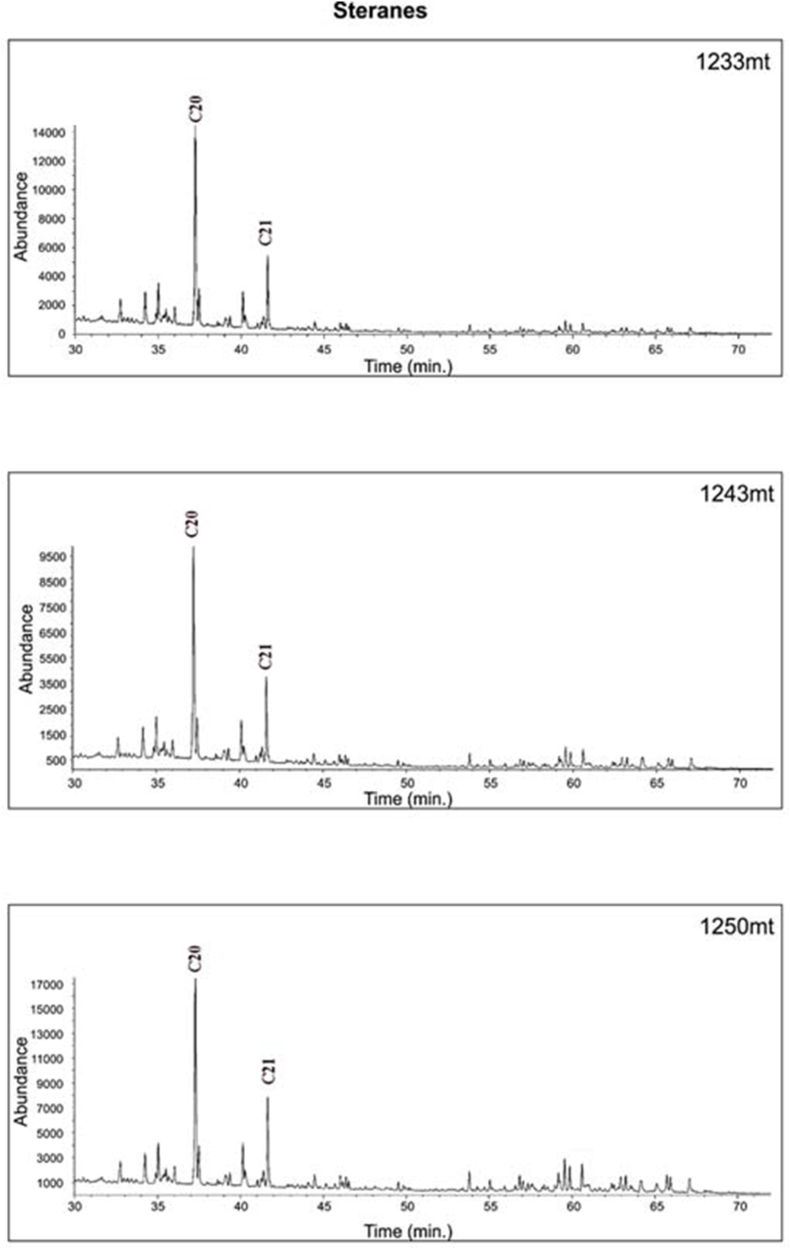
GC-MS *m*/*z* 217 Chromatograms for Akkas-3 Samples. See Fig. 4 for sample locations.

**Fig. 20:**
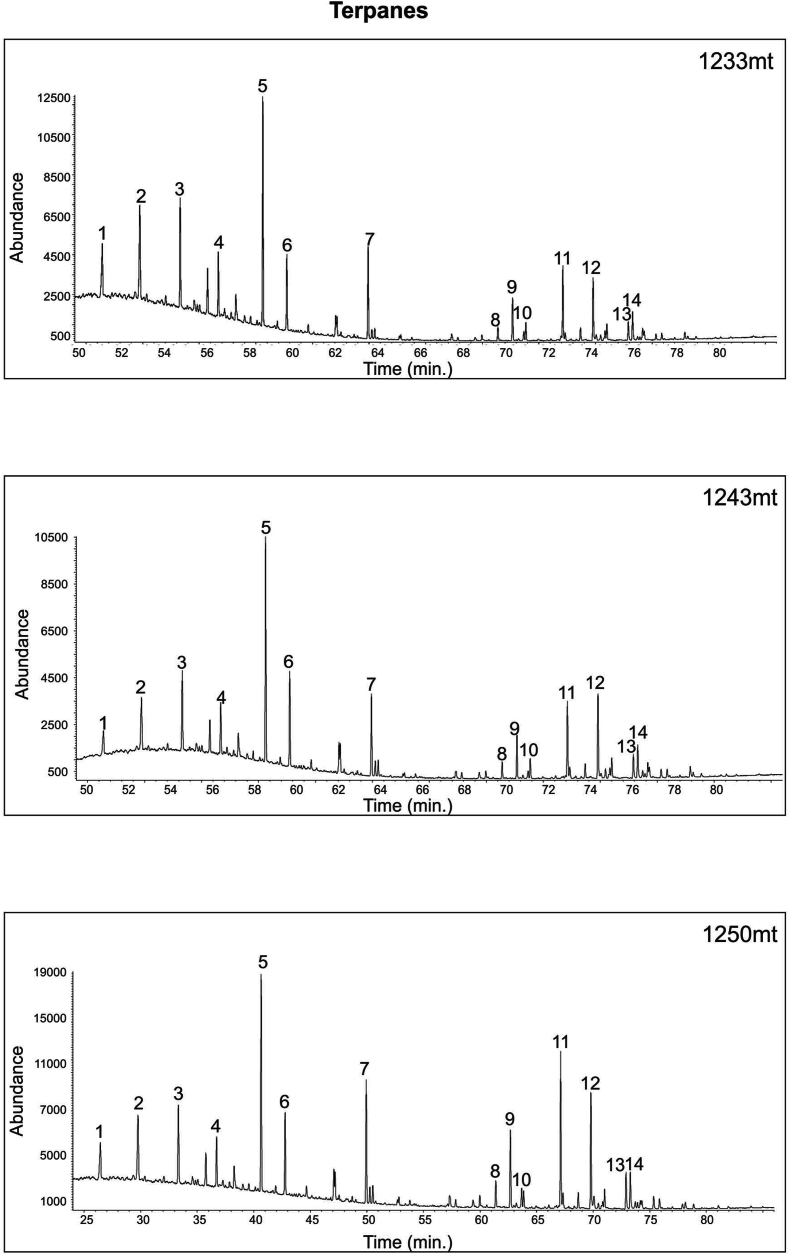
GC-MS *m*/*z* 191 Chromatograms for Akkas-3 Samples, See Fig. 4 for sample locations.

The results of the study of three extracts using gas chromatography-mass spectrometry for steranes (*m*/*z* 217) and hopanes (*m*/*z* 191) illustrated in Fig. 19, Fig. 20 and Table 6). Moreover, C_30_ steranes (24-n-propylcholes-tane) are an indication that marine algae have been produced [61] (Fig. 20), and carbon isotope values all indicate marine organic matter (Table 3 and Fig. 14).

**Table 6:** Peaks identification of Tricyclic and Pentacyclic terpanes.

No.	Code	Biomarker ID	*m*/*z*
1	C19T	C19H34 tricyclic diterpane	191
2	C20T	C20H36 tricyclic diterpane	191
3	C21T	C21H38 tricyclic diterpane	191
4	C22T	C22H40 tricyclic terpane	191
5	C23T	C23H42 tricyclic terpane	191
6	C24T	C24H44 tricyclic terpane	191
7	TET	C24H42 teteracyclic terpane	191
8	Ts	18α, 21β-22,29,30-trisnorhopane	191
9	Tm	17α,18α,21β-25,28,30-trisnorhopane	191
10	C30S	C30H56 tricyclic terpane	191
11	C29H	17α, 21β-30-norhopane	191
12	C30H	17α, 21β-hopane	191
13	C31S	17α, 21β-30-homohopane (22S)	191
14	C31R	17α, 21β-30-homohopane (22S)	191

**Fig. 21:**
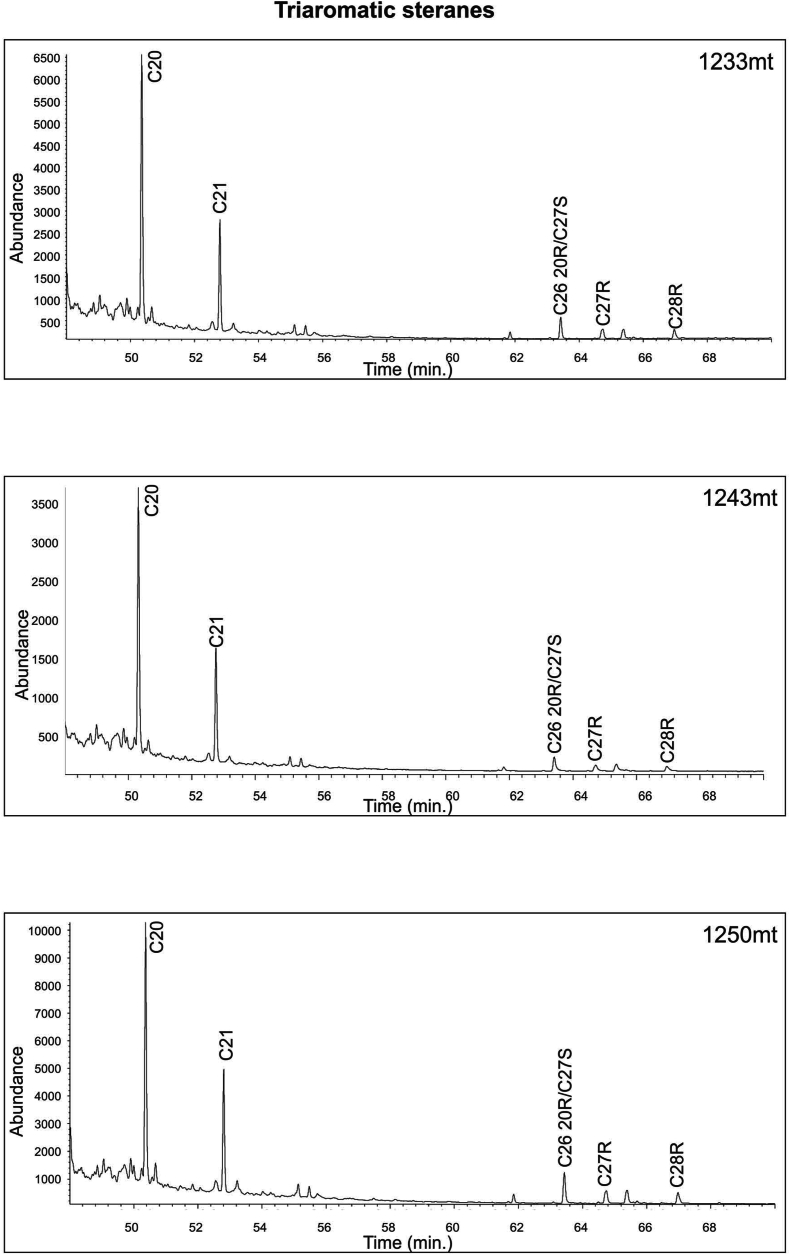
GC-MS *m*/*z* 231 Chromatograms for Akkas-3 Samples. See Fig. 4 for sample locations.

The low C_24_/C_23_, high C_22_/C_21_ tricyclic terpanes (Table 5) [62], high C_27_ diasteranes/regular steranes (range 0.75–0.86; average 0.79; Table 5), all support the shale source rock idea. On the other hand, these samples have high C_29_/C_30_ hopane ratios (0.91–1.35) (Table 5). These high ratios may be due to high thermal maturity level (see below) which led to continuously destruction of the high molecular weighty compounds to low molecular weight ones. These values indicate oxic marine environment conditions [53] (Fig. 21).

The determination of redox conditions can also be inferred by examining the presence of pristane and phytane in the samples under investigation. Pristane is typically linked to oxidizing conditions, while phytane is commonly associated with reducing conditions [63]. Consequently, a high Pr/Ph ratio signifies the presence of oxic conditions, whereas a low ratio indicates anoxic conditions. In the current samples, the values of pristine/phytane were found to be less than one (Table 4), and this is evidence of reducing conditions of the corresponding source rocks [64]. According to pristine/phytane values, the Ora Formation in Akkas oilfield, western Iraq, was deposited under an oxic depositional environment [16]. As previously indicated, Ora Formation was deposited in fluvial-delta environments. These depositional environments are usually characterized by oxic-dysoxic conditions. Moreover, as noted above, the studied samples are contaminated by hydrocarbons; therefore, the low Ph/Ph values from the present study may represent the paleodepositional conditions of the source rocks which generated these hydrocarbons (light oil and condensate) in the Akkas field. On the other hand, the C_35_S/C_34_S values are low (average C_35_S/C_34_S is 0.63, Table 5). These low values may be due to maturity effect, where this ratio decreases with maturity [53].

### 4.4 Thermal maturity

Determining the thermal maturity of lower Paleozoic shales poses challenges as vitrinite formation was limited due to the absence of land plants during that time [[65], [66], [67]]. Therefore, in this study, Tmax (maximum temperature) and biomarker maturity parameters were employed to evaluate thermal maturity level of Ora Formation (Tmax) and the contaminant (biomarker). The Tmax values of all samples exhibiting relatively high S2 (greater than 1 mg HC/g TOC) are below 430 °C (425–428 °C) (Table 1). Based on these values, the organic matter within these shales of Ora Formation is immature. Conversely, the biomarker ratios are shown to be highly inconsistent with Tmax values as they indicate high maturity levels. This likely indicates that the biomarker ratios rather represent the maturity level of the contaminant hydrocarbons.

The high maturity level is evidenced by the heightened values of the tricyclic terpanes/hopanes ratio, ranging from 1.65 to 3.17 as detailed in Table 7. This ratio tends to increase with maturity, as more tricyclic terpanes are generated than hopanes at higher maturity levels, as explained by Ref. [68]. This elevated maturity level is further affirmed by the pronounced relative abundance of pregnane and homopregnane (C_21_ and C_22_ sterane) compared to the normal steranes (Fig. 19). In addition, strong indications of high maturity include the decay or semi-vanishing of regular sterane and a clear predominance of pregnane and homopregnane (C_21_ and C_22_ sterane) [69] (Fig. 19), in the Akkas-3 samples. Moreover, the high diasteranre/sterane values (0.75–0.86) is another indication for the high maturity level; where diasteranes are more resistant than regular steranes to thermal degradation.

**Table 7:** Thermal maturity biomarker ratios for the Akkas-3 samples.

Well	Depth (m)	Ts/Ts + Tm	C_32_ 22S/(S + R)	C_29_S/(S + R)	C_29_ββ/(αα+ββ)	M/H	R0 E	VRc%	MDR	MPI	4MDBT/1MDBT	TAS(CR)	tricyclic terpanes/hopanes
Akkas-3	3759	0.25	0.47	0.51	0.44	0.26	0.81	0.544	2.90	0.69	2.90	0.82	3.17
Akkas-3	3789	0.28	0.49	0.45	0.43	0.24	0.94	0.49	2.51	0.90	2.51	0.85	2.42
Akkas-3	3810	0.26	0.55	0.61	0.52	0.17	0.87	0.544	2.81	0.79	2.81	0.76	1.65

Aromatic hydrocarbons are commonly employed for assessing thermal maturity levels. The Methylphenanthrene Index values (MPI) according to Ref. [70] for the three samples in Table 7 fall within the range of 0.69–0.90. The vitrinite reflectance values calculated based on MPI also align with the earlier findings, ranging from 0.81 to 0.94 % RoE. Another indicator, the Methyldibenzothiophene ratio (MDBT = 4-/1-MDBT), proposed as a more robust maturity index with a strong correlation to Tmax in source rock bitumen and vitrinite reflectance, tends to increase with maturity, especially at advanced stages of thermal transformation [71]. In this study, the MDBT values for the samples range from 2.50 to 2.90 (Table 7), suggesting a high thermal maturity level. Furthermore, the Triaromatic Steroids Cracking Ratio (CR) serves as a reliable maturity indicator compared to steranes and hopanes. This ratio remains independent of lithology, depositional environment, and type of organic matter, being affected solely by maturity. Consequently, it is valid for assessing highly mature organic matter. As maturity increases, the abundance of long-chain triaromatic steroids (C26–C28) decreases relative to short-chain ones (C20–C21) due to the higher resistance of short chains to thermal degradation compared to long chains [53] or the cracking of long chains generating short-chain triaromatic steroids [72]. The CR values for the studied samples fall within the range of 0.76–0.85 (Table 7), indicative of a high maturity level.

### 4.5 Probable source rocks and regional significance

The lower Paleozoic successions have two potential source rocks; the first is the lower Silurian hot shale of Akkas Formation, and the second is shale of the Khabour Formation [14,27,73]. The two candidate source rocks are black shales of the lower Silurian hot shales contain mainly marine organic matter (type II kerogen) and were deposited under reducing conditions. In Akkas-1 well, the hot shales of Akkas Formation have TOC values between 2.6 and 6.97 wt%; whereas the TOC values of Khabour Formation in the range of 0.11–1.1 wt% [51], and may up to 5 % [17]. The organic matter of the hot shales is immature to early mature, and they suggested to be deposited under euxinic conditions [74], but they may be highly mature in other area. Organic matter of Khabour Formatoin is mature to highly mature, and the lower part of the Khabour Formation consists of highly mature organic-rich black shale [51]. Therefore, Khabour Formation is most likely the source rocks for these hydrocabons in the middle Paleozoic (late Devonian-early Carboniferous Ora Formation.

In terms of the regional context, the Ora Formation may be correlated with the Markada or Doubayat Formation in Syria, the Koprulu Formation in Turkey, and the upper part of the Jubah Formation in Saudi Arabia. The Jubah Formation in Saudi Arabia consists of continental clastics, which are replaced by mixed marine siliciclastics and carbonates in southeast Turkey and northern Iraq. The dominance of marine environments, particularly during the latest Devonian in the northern region, indicates a potential differential downwarp of the northern margin of Gondwana. This could imply that the northern margin of Gondwana became tectonically unstable due to the onset of the Hercynian Orogeny [75,76].

In Iraq, the maximum flooding surface (MFS D30) was positioned in the shale of the Ora Formation [21], which also shows good correlation with the MFS D30 of the Koprula Shale in south Turkey and Jubah Formation in south parts of the Arabian Plate. This correlation suggests that the Arabian Plate was regionally flooded in late Devonian-Mississippian [21,[77], [78], [79]]. Maximum flooding surface (MFS) are horizons of maximum transgression within a sequence, this transgression can cause various salinization processes, which in effect preserve organic content in an anoxic environment and increase the potential of the source rocks [80].

## 5 Conclusions

Organic geochemical analyses have been performed on the shale and muddy limestone succession of the Ora Formation, encompassing both surface and subsurface sections in the northernmost and western regions of Iraq. These analyses involved the utilization of Total Organic Carbon (TOC) and HAWK pyrolysis techniques, supported by X-ray diffraction and scanning electron microscopy investigations, gas chromatography, and gas chromatography-mass spectrometry are utilized.

The TOC and HAWK results indicate a moderate to high level of organic enrichment within the Ora Formation. The HAWK data obtained from surface samples suggest that they are either highly mature, highly weathered (oxidized), or both. Conversely, the subsurface samples exhibit a mixed composition of kerogen types III and II-III, with the organic matter being immature. High clastic input is one of the elements that contributes to the production of organic-lean sediments, sedimentation rate, prevailing oxic environment during deposition, and the effects of weathering.

The observed paleoenvironmental conditions are supported by the common presence of detrital influx of quartz and feldspars in the studied samples. Furthermore, the dominance of oxic conditions in surface and subsurface samples; with some interval deposited under anoxic conditions as demonstrated by the occurrence of pyrite. Clay minerals such as illite and kaolinite provide more evidence that the Ora Formation was deposited under hot arid, and warm humid paleoclimatic conditions. Subsurface samples of the Ora Formation's light hydrocarbons were analyzed using gas chromatography and gas chromatography-mass spectrometry. The results showed that these hydrocarbons are very mature and probably came from shale source rocks that included organic materials from marine planktonic algae. These shale rocks formed in anoxic environments at sea. The rocks from which these hydrocarbons likely originated are located in the lower section of the Khabour Formation.

## Data availability statement

Data relevant to study is not kept in publicly accessible repositories; instead, it is provided upon request.

## CRediT authorship contribution statement

**Al-Auqadi Rahma S:** Conceptualization. **Mohamed W. Alkhafaji:** Investigation. **Ali I. Al-Juboury:** Investigation. **Alex Zumberge:** Investigation, Formal analysis. **Nasir Alarifi:** Funding acquisition. **Dan Jarvie:** Data curation. **Giovanni Zanoni:** Investigation. **Harry Rowe:** Formal analysis.

## Declaration of competing interest

The authors declare that they have no known competing financial interests or personal relationships that could have appeared to influence the work reported in this paper.
